# The Genome of Polymorphonuclear Neutrophils Maintains Normal Coding Sequences

**DOI:** 10.1371/journal.pone.0078685

**Published:** 2013-11-08

**Authors:** Fengxia Xiao, Yeong C. Kim, Hongxiu Wen, Jiangtao Luo, Peixian Chen, Kenneth Cowan, San Ming Wang

**Affiliations:** 1 Department of Genetics, Cell Biology and Anatomy, University of Nebraska Medical Center, Omaha, Nebraska, United States of America; 2 Department of Medicine, College of Medicine, University of Nebraska Medical Center, Omaha, Nebraska, United States of America; 3 Department of Biostatistics, College of Public Health, University of Nebraska Medical Center, Omaha, Nebraska, United States of America; 4 Fred & Pamela Buffet Cancer Center, University of Nebraska Medical Center, Omaha, Nebraska, United States of America; University of Kentucky, United States of America

## Abstract

Genetic studies often use genomic DNA from whole blood cells, of which the majority are the polymorphonuclear myeloid cells. Those cells undergo dramatic change of nuclear morphology following cellular differentiation. It remains elusive if the nuclear morphological change accompanies sequence alternations from the intact genome. If such event exists, it will cause a serious problem in using such type of genomic DNA for genetic study as the sequences will not represent the intact genome in the host individuals. Using exome sequencing, we compared the coding regions between neutrophil, which is the major type of polymorphonuclear cells, and CD4+ T cell, which has an intact genome, from the same individual. The results show that exon sequences between the two cell types are essentially the same. The minor differences represented by the missed exons and base changes between the two cell types were validated to be mainly caused by experimental errors. Our study concludes that genomic DNA from whole blood cells can be safely used for genetic studies.

## Introduction

Genomic DNA from peripheral blood cells is routinely used for genetic studies. For example, it is a common practice to use blood DNA to distinguish germline variation and somatic mutation in solid tumors [1]–[7]. Blood cells consist of multiple cell types of myeloid and lymphoid lineages [8]. Myeloid cells are differentiated rapidly from myeloid progenitors to myeloblasts and to mature terminal cells of neutrophils, eosinophils, basophils and monocytes, towards the end stage of cellular destruction by apoptosis, necrosis or netrosis [9]–[10]. During differentiation, the nuclei of myeloid cells transform from mononuclear to segmented and banded polymorphonuclear shape of 2–5 lobes. Little is known if the nuclear morphological transformation during myeloid differentiation accompanies any genome sequence change. If such change does exist, the sequences derived from blood cells containing myeloid cells will reflect the genomes of mixed mononuclear and polymorphonuclear cells. Interpretation of such heterogeneous sequences will be problematic. While studies on selected genes were performed [11], no systematic attempts have been reported to determine, at genome level, the nature of genetic sequences in polymorphonuclear myeloid cells. In this study, we used the exome sequencing method [12] to analyze the entire coding regions of neutrophils, the most abundant myeloid cells constituting 40–60% of nucleated cell counts in peripheral blood [9], and compared the data with the mononuclear CD4+ T cells represeenting the intact genome of the same individual. Our study shows that the coding regions in the polymorphonuclear neutrophils are essentially the same as the intact genome.

## Results and Discussion

Using exome sequencing method, we analyzed the coding regions between the genomes of polymorphonuclear neutrophils and mononuclear CD4+ T cells from the same healthy individual. our study detected 197,988 (98.5%) and 197,565 (98.3%) of the targeted 201,046 human genome exons in neutrophils and in CD4+ T cells respectively, of which 196,749 exons are the same between the two cell types (Table 1). And there are 3,058 and 3,481 (1.5% and 1.7%) exons missed in neutrophils and CD4+ T cells respectively, of which 2,242 are missed in both cell types and the rest are missed in a single cell type (Table 1). Furthermore, there are 150,719 SNVs detected in neutrophils and 150,203 SNVs in CD4+ T cells, of which 141,034 are common between the two cell types, and 9,685 (6.4%) and 9,169 (6.1%) are only present in neutrophils and CD4+ T cells, respectively (Table 2).

**Table 1:** Exome data collected from neutrophils and CD4+ T cells.

items	Neutrophils	CD4+ T cells
Total sequenced bases	14,686,433,331	13,784,953,286
Exome coverage	237	222
Targeted exon by exome kit	201,046 (100%)	201,046 (100%)
Captured exon by exome sequencing	197,988 (98.5%)	197,565 (98.3%)
Exons common in neutrophils and T cells	196,749 (97.9%)	196,749 (97.9%)
Missed exons by exome sequencing	3,058	3,481
Missed in both cell types	2,242	2,242
Missed in neutrophils	816	–
Missed in CD4+ T cells	–	1,239

**Table 2:** SNVs detected in neutrophils and CD4+ T cells.

Cell type	SNV	SNV distribution	
		Common in both cell types	Only in single cell type
Neutrophils	150,719 (100%)	141,034 (94%)	9,685 (6%)
CD4+ T cells	150,203 (100%)	141,034 (94%)	9,169 (6%)

**Figure 1:**
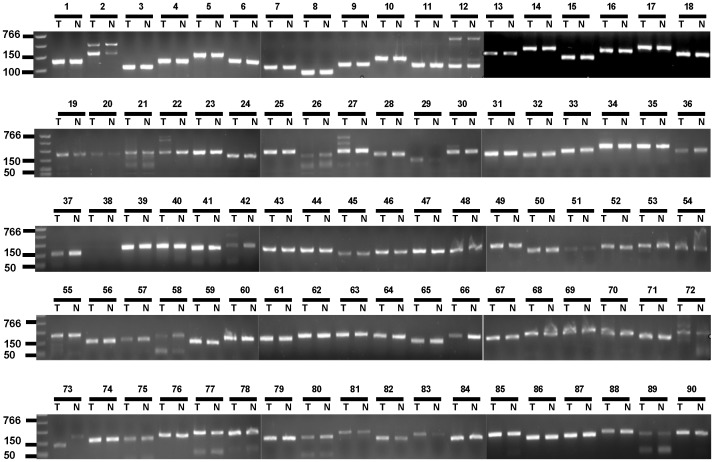
PCR detection of the missed exons. One hundred of missed exons were selected for the validation and 90 generated positive results. Except #73, all 89 reactions detected the missed exons with the same sizes between T cells and neutrophils. T: CD4+ T cells; N: neutrophils.

We used PCR to test if the missed exons reflect the true exon differences or were caused by experimental artifacts. Based on statistical analysis, we randomly picked 100 missed exons for the validtion, which provide 88% of probability to test a missed exon. Three types of results were generated: 1) 89 reactions detected the targeted exons with the same size in both T cells and neutrophils, implying that those missed exons are present in both T cells and neutrophils (Figure 1); 2) 10 reactions failed to detect the missed exons in both T cells and neutrophils, implying that those exons may not be present or may not be included in the exome kit-targeted exons in this donor’s genome; and 3) 1 reaction (#73 in Figure 1) detected the missed exons with different size in T cells and neutrophils. This exon is for *AIRE*, a gene involved in regulating auto-antigen expression and auto-reactive T-cell negative selection. The results indicate that most of the exons missed from exome data were caused by experimental failure, likely missed during exome DNA capturing process, an event often present in exome sequencing study [13]. We then used Sanger sequencing to validate if the observed single-base differences between T cells and neutrophils reflect the true variants bewtween the two cell types, or if the differences were generated by sequencing errors or miscalling. Based on statistical analysis, we selected 40 candidates for validation, which provides 91.6% probability to confirm a variant. Of the 39 successful reactions, 18 are determined as sequencing errors, 8 are confirmed as true homozygous variants in the individual genome, and 13 are validated as heterozygous variants but misinterpreted by mapping program (Table 3). Therefore, the variants mapped differently between T cells and neutrophils are mostly caused by sequencing errors or miscalling in the mapping process. TCR loci in T cells can be highly polymorphistic due to VDJ recombination. We compared the sequences from neutrophils and T cells mapped to the TCR-related loci (TCR-alpha and TCR-delta, chr14∶22,205,021-23,021,097; TCR-beta, chr7∶142,000,946-142,945,186; TCR-gamma, chr7∶38,288,844-38,403,119; and PTCRA, chr6∶42,883,727-42,893,575), but we did not find any coding differences for these loci between the two cell types. We also compared the exome data between neutrophils and CD19+ B cells of the same individual, and also not observed any diffferences (data not shown).

**Table 3:** Sanger sequencing validation for single-base differences between neutrophils and T cells.*

Position	hg19	Mapped variants		Validation	
		T	N	T	N
Sequencing error					
chr1∶148252811	T	A	wt	T	T
chr2∶174939985	G	T	wt	G	G
chr9∶68455295	C	A	wt	C	C
chr12∶123893145	C	T	wt	C	C
chr16∶31002833	G	A	wt	G	G
chr16∶72763868	C	A	wt	C	C
chr17∶58286681	G	C	wt	G	G
chr19∶58002709	C	T	wt	C	C
chr20∶26258754	T	G	wt	T	T
chrY:10028388	A	G	wt	A	A
chr1∶143398955	T	wt	C	T	T
chr5∶49440947	C	wt	G	C	C
chr16∶19502080	C	wt	T	C	C
chr17∶7733570	T	wt	G	T	T
chr21∶9650474	G	wt	T	G	G
chrX:123199635	G	wt	T	G	G
chrY:28780697	G	wt	A	G	G
chrY:28780699	C	wt	G	C	C
Homozygous SNVs					
chr4∶170990053	C	T	wt	T	T
chr9∶3270683	C	T	wt	T	T
chr14∶105270485	G	T/+T	wt	+T	+T
chr17∶48313505	G	T	wt	T	T
chr10∶33134724	T	wt	C	C	C
chr10∶30305222	T	wt	G	G	G
chr11∶120338142	C	wt	T	T	T
chr14∶24887303	A	wt	C	−A	−A
Heterozygous SNVs					
chr1∶2980578	C	T	wt	C/T	C/T
chr5∶137290214	T	C	wt	T/C	T/C
chr8∶139160613	A	G	wt	A/G	A/G
chr17∶36333536	A	C	wt	A/C	A/C
chr22∶25006880	G	A	wt	G/A	G/A
chr1∶149724356	G	wt	A	G/A	G/A
chr3∶111577836	A	wt	T	A/T	A/T
chr3∶189713346	T	wt	G	T/G	T/G
chr3∶195295510	A	wt	G	A/G	A/G
chr15∶20867219	A	wt	G	A/G	A/G
chr19∶55221804	G	wt	+AC	G/C	G/C
chr19∶55281149	C	wt	T	C/T	C/T
chr21∶10897305	C	wt	T	C/T	C/T

* wt: wild type; +: insertion, −: deletion, /: heterozygote.

Myeloid cell lineage undergoes rapid differentiation and dramatic nuclear morphological change. While it remains unknown if any sequence changes in the non-coding regions could occur during myeloid differentiation and certain very rare mutations can exist in the coding regions [14], our study shows that the coding genes in polymorphonuclear neutrohophils remain essentially the same as the intact genome. Our study suggests that the chromosomes in myeloid cells remain linear chromatin structure regardless the morphological changes during myeloid differentiation. Our study concludes that genomic DNA from myeloid lineage cells can be safely used in genetic studies.

## Methods

Ethics Statement: The cells used for the study were obtained from AllCells LLC (http://www.allcells.com/, Emeryville, California), which has its full IRB system (Biomed IRB) for providing human blood cells from donors for research. The donor signed the written consent form, which is archived with their medical records. According to US Federal Regulations, 45 CFR Part 46.101(b)(4)–Protection of Human Subjects, using this type of human cells for research is exempted from the requirement for IRB review. Peripheral leukapheresis blood was collected from a healthy Caucasian male donor, with cell count of red blood cells of 4.76×10^3^/mm^3^, and leukocyte differentiation of lymphocytes 1.7×103/mm^3^ (23.0%), monocytes 0.3×10^3^/mm^3^ (4.7%), and granulocytes of 5.8×10^3^/mm^3^ (72.3%). The collected blood sample was used immediately for cell purification: red blood cells were depleted by sedimentation using the HetaSep solution (Stem Cell Technologies); neutrophils were isolated by using the EasySep human Neutrophil Enrichment Kit (Stem Cell Technologies); mononuclear cells were isolated by using Ficoll solution (GE Healthcare) and CD4+ helper T cells were isolated from the mononuclear cells using the StemSep human Naïve CD4+ T Cell Enrichment Kit (Stem Cell Technologies). The purity of the isolated cells was determined by FACS analysis with 90% for neutrophils and 97% for CD4+ T cells. DNA was extracted from the purified cells by using the FlexiGene DNA kit (QiaGen).

Exome DNA was capyured from DNA sample of neutrophils and CD4+ T cells using Illumina TruSeq exome enrichment kit following manufacturer’s protocols (http://www.illumina.com/products/truseq_exome_enrichment_kit.ilmn). Paired-end exome sequencing (2×100) was performed at 200x exome coverage per sample using an Illumina Hiseq 2000 sequencer. Sequences from each cell type were compared to the 201,046 exons covered by the Illumina TruSeq exome enrichment kit (Illumina TruSeq Exome Targeted Region database 1.3.0). Exome sequences were mapped to the human genome reference sequences (hg19) using BWA-SW [15] and SAMtools [16] with default parameters. Variations were called using VarScan 2 [17] on the conditions of minimum coverage >10, minimum variation frequency >0.2, minimum average quality score >30, and p-value <0.05. The called variations were searched in dbSNP135 for SNP identification.

PCR and Sanger sequencing were used to validate the missed exons and single-based variants identified by exome mapping. We performed a statistical analysis to determine the proper number of candidates for the validations. Assuming the missed exons and variants occur at random with probability , the number of the missed exons and variants in sequenced candidates will follow a binomial distribution . Then the probability of at least missed exons and variants is

where represents binamial random variable, represents minimal number of missed exons or variants, represents binomial distribution, represents total sample size. The rate of missed exons is 0.021. Testing 100 candidates will provide 0.880 chance to detect each missed exon; the rate of single base variants is 0.06. Testing 40 candidates will provide 0.916 chance to detect a variant [18].

PCR primers were designed by Primer3 (http://frodo.wi.mit.edu/primer3/). PCR was performed with DNA (20 ng/reaction), sense and antisense primers (10 pmol), and GoTaq® DNA polymerase (1.25 unit, Promega) at the conditions of denaturing at 95°C 7 minutes, 37 cycles of 95°C 30 seconds, 57°C 30 seconds, 72°C 45 seconds, final extension at 72°C 7 minutes. PCR products were checked on 2% agarose gels. For these to be sequenced, each was purified using an Illustra GFX 96PCR Purification kit (GE Healthcare), and sequenced using BigDye Terminator v3.1 in an ABI3730 DNA sequencer (Applied BioSystems).

The exome data from neutrophils and CD4+ T cells have been deposited in NCBI, pending for assigned accession. The exome data from neutrophils and CD4+ T cells have been deposited in NCBI, with accession number SRR933550 for Neutrophils and SRR933549 for CD4+ T cells.
